# Medical Jurisprudence

**Published:** 1825-07-01

**Authors:** 


### Medical Jurisprudence.

1. *Charge of Poisoning by Arsenic.* Messrs. Orfila, Vauquelin, and Barreul, were summoned by the Court of Assize, Department of the Aub, to ascertain whether certain matters found in the possession of the widow Laurent, contained arsenic. This widow had been accused of poisoning her husband ten days after marriage. The medical men who had examined the husband's body, came to the conclusion that the deceased was poisoned by arsenic; while, on the other hand, numerous experiments made at the laboratory of the Faculty of Medicine of Paris, could detect no arsenic in the matters submitted for analysis. The magistrates, however, were not satisfied, and M. Orfila was brought into court, when the following questions were put to him.

1st. Did the experiments made by those who first examined the body, prove that the matters taken from the stomach of Laurent contained arsenic?

2d. Is it possible to find, in the digestive canal of a person not killed by arsenic, grains, in appearance, of that mineral?

3d. What conclusion can be drawn from the circumstance of a fowl dying after eating of the barley of which the deceased's ptisan was prepared?

4th. What conclusion can be drawn from the death of the leeches applied to the epigastrium of the deceased?

5th. Can it be concluded from the symptoms during the life and appearances on dissection of the deceased, that he died of poison?

1. *Answer to 1st Question.* The physicians charged with examining the body, in the first instance, state that they found in the stomach of Laurent a *pulverulent* substance which, when thrown on burning charcoal, *detonated*, *burned with a flame*, and *emitted the odour of garlic*. They hesitated not, therefore, to declare that the substance was white oxyde of arsenic. This conclusion was erroneous, for the oxyde of arsenic does not possess the property of detonating nor burning with a flame, when thrown on burning charcoal. As to the smell of garlic, it is not conclusive, since it might result from other substances than garlic, which might happen to be in the stomach. They should have dissolved the suspected substance in boiling water, and shewed that a *green* precipitation took place on adding sulphate of ammoniacal copper, and a *yellow*, on the addition of hydro-sulfuric acid. This last precipitate ought also to have dissolved rapidly in liquor of ammonia.

2. *Answer to 2d Question.* The stomach of the deceased might have contained a granular substance possessing most of the properties assigned by the physicians to the supposed arsenical grains. Under certain circumstances, it is observed that the mucous membrane of the stomach and bowels is lined with a multitude of shining points, composed of fat and albumen. These, when thrown on burning charcoal, decrepitate while drying, and go off with a noise which might be designated (though not properly,) detonation. These little bodies inflame like fatty matters, and exhale an effluvium of burnt animal substances. That these appearances are liable to lead to errors of a serious nature, the following instance is related to prove:—

On the 2d of August, 1824, the exhumation of an individual, 38 years of age, and who had been buried six weeks, was ordered by the *Procureur du Roi*. The lower extremity of the œsophagus, and the mucous membrane of the stomach and duodenum were found inflamed. A number of the small bodies, described above, were found in the digestive tube, and were considered by the medical man appointed to analyse them, as oxyde of arsenic altered by animal matters. The substances, however, were transmitted to Paris, and analysed by M. Vauquelin, who decided that they consisted of animal matters only, and contained no mixture of arsenic. Another instance, of a similar kind, is also stated by M. Orfila.

3. *Answer to Question three.* It is to be remarked that Laurent died five days after eating an omelette, in which was the suspected poison. In this interval, he had taken several ptisans prepared by his wife. One of the physicians who visited the deceased, struck with the disagreeable savour of the ptisan, gave some of the grains of barley in it to a fowl, which fowl died the day following. A cat, who had devoured the entrails of the fowl, was seized with violent convulsions. These were puzzling facts, and we shall give M. Orfila's answer, verbatim.

"When oxyde of arsenic is boiled with barley in water, the mineral is dissolved, and the liquid is rendered poisonous. The grains of barley, during this process, swell and imbibe the arsenical solution, and, when given to fowls, destroy them. But, on the other hand, if the barley water be first prepared, and then the arsenic infused and dissolved in it, the grains of barley (having been swelled previously to the addition of the poison,) will not imbibe any more of that fluid, and consequently will be innocuous to fowls, *provided no grains of undissolved arsenic adhere to the barley.* These facts and considerations justify the following answer, which I gave to the question, namely. The arsenical oxyde which, according to the act of accusation, was not introduced into the barley-water till after the latter was prepared, would remain dissolved in the fluid—and the barley itself would not be poisonous—*provided no particles of undissolved arsenic adhered to the barley.*"*

4. *Answer to Question four.* The circumstance of the leeches dying soon after coming off the epigastrium, is quite undeserving of any serious answer. Every body knows that these animals frequently die immediately after being applied to the bodies of patients, whatever may have been the nature of the maladies under which they laboured.

5. *Answer to Question five.* Laurent certainly evinced symptoms similar to those resulting from the exhibition of white oxyde of arsenic; but then there are numerous affections, in which we observe a similar analogy of symptoms, and where there can be no suspicion of poison. The ensemble of the phenomena, in the case under consideration, render it *probable* that poison had been administered. There are many instances on record where, although arsenic was actually administered, the usual symptoms did not follow—and vice versâ. Hence, where the mineral itself can be detected, the absence of the usual symptoms is no proof against the administration of the poison—while, on the other hand, the presence of the symptoms, without the actual detection of the arsenic, can afford no sufficient proof of poison having been given.

In illustration of the uncertainty attending the post-mortem appearances in cases of poison, M. Orfila stated to the court the following very curious history.

" M———, aged 45 years, swallowed, in a paroxysm of passion, at eight o'clock in the morning, three drachms of white arsenic diffused in a glass of water. He immediately disclosed the transaction to one of his female friends, and rushed out of his house. Some of the arsenic remained in the glass, and was ascertained as such. The gentleman was searched for, in vain, during the space of two hours. At ten o'clock he voluntarily made his appearance. The imminent danger of his situation was represented to him—he acknowledged again that he had swallowed arsenic—and consented to take an emetic of tartarized antimony. The emetic had no operation. Large quantities of milk

* With all due deference to M. Orfila, we think this was giving no satisfactory evidence at all. How could the court tell whether or not the fowl was poisoned by arsenic sticking to the barley?

and mucilaginous drink were next administered, which had the effect of evacuating the greater proportion of the ingesta which had been taken. In one hour after this (at 12 o'clock, or later,) the patient, who had previously suffered little or no inconvenience, complained of a painful sense of constriction at the epigastrium, accompanied by burning heat and thirst. His features became altered—the pulse was quickened. These symptoms became more and more intense—the parietes of the abdomen were drawn towards the spine—the pulse became small and intermittent—and at five o'clock in the afternoon he expired.

" *Dissection.* No effusion in the abdomen—all the viscera of this region were in a natural state. The mucous membrane of the stomach and bowels presented no trace of redness, inflammation, or any change of structure. Oxyde of arsenic was found in the stomach and duodenum, in considerable quantity."

Such was the evidence given by M. Orfila. The widow of Laurent was acquitted.—GAZETTE DE SANTE.

---

2. *Contagion—Plague—Quarantine.** Our sentiments respecting *positive* contagion in plague, and *contingent* contagion in fevers, have been long before the public, and need not now be reiterated. The dream of the Anticontagionists is vanished—their " fool's Paradise" is dissolved into air. The Foreign Secretary's late speech in the House of Commons, will not indeed cure them of their dear *delusion*—but it will leave them with a small train of their deluded disciples. They need hardly quarrel now for the honour of priority in radiating the new light which, after all, is but old darkness rendered visible by medical fanaticism. Mr. Canning has put an extinguisher on the *ignis fatuus* of the Macleananites. Their wild theories—bold assertions—sophistical reasonings—distortions of facts—historical perversions—and last, not least, their proud vapourings, have made very few converts among the only legitimate judges,—their medical brethren. Their commercial masters, for whom they worked so hard, have now been rightly served. The quarantine laws are exasperated abroad, in tenfold proportion to their mitigation at home! For these shackles on commerce our merchants are entirely indebted to the raving red-hot zeal and confident predictions of the Macleanean dogmatists. We need hardly say that the sole drift of the mercantile Anticontagionists was—*self-interest. Their* science consists exclusively in the knowledge of numbers—the balance of profit and loss. The moment the doctrine of Maclean and his disciples produces results on the wrong folio of their ledgers (and this is probably the case already,) the city merchants will be as stanch contagionists as Sir Gilbert himself. It will soon be—" Quarantine for ever!—down with Macleananism!"

From such self-interested declaimers, we turn with sorrow, to a few—a very few, who have joined in the mercantile clamour for other and

* Dr. Macmichael—Dr. Granville—Dr. Larkin—The Westminster Review—Dr. Armstrong's lectures, cum multis aliis.

more honourable reasons. Entangled in the mazes of visionary enthusiasts we grieve to see an Armstrong! That we should be obliged to differ from him in opinion, we sincerely regret—but that he should so frequently differ from himself, is far more to be regretted. We blame no man for altering his opinions—we honour every man for a candid avowal of such alterations. But the extremes of tenacity and mutability are dangerous. Every man should be open to conviction—but that conviction should not be too hastily admitted or avowed, nor too frequently changed or modified. Dr. Armstrong is doing himself more harm than all his enemies collected could do. He is committing *felo de se* on his own well-earned reputation. If no other friend will have the courage or candour to warn him of his danger, we will do so. We have now planted a beacon on a hidden rock that lies in the very middle of that rapid stream of renown on which his bark is carelessly careering!

Mr. Larkin, too, a young gentleman of promise, has early suffered a bias, and become infected with the erroneous doctrines of the day. His mind, however, is immatured by time or experience, and better things may be expected from him at a future period. Meanwhile, he has wasted the sweets of learning on the desert *air*, for the contagion of plague is just where it was. We may admire the ingenuity, the zeal, the research, and the eloquence of a man endeavouring to sweep away *facts*, and place in their stead the phantoms of an ardent imagination; but, like the rocks of the ocean, these facts rise again unchanged, as soon as the tide of oratory has subsided. Mr. Larkin says, he could have piled Pelion on Ossa,* to prove that the plague is not contagious. Well! we suppose we must not question the mountain moving power of Mr. Larkin's arm; but we may be permitted to express our opinion, that had he piled Pelion on Ossa, he would have been just as far from his object as the giants were from the skies. They failed in scaling the Heavens—he has failed in disproving the contagion of plague. "The doctrine (of contagion in plague,) says Mr. Larkin, is in itself so improbable, that it carries its own refutation with it; and those who support it, I regard in no other light than that of mere alarmists." This is strong language, when we consider that it is directed by a youth against 99 in a hundred of his professional brethren, not only in this, but in every country of the world. It is quite unnecessary now to enter at all into the question. Nothing new has been elicited of late on either side. The great mass of medical society remain firm in that belief which has been founded for ages on the evidence of facts; while the legislature of the country has openly and unequivocally disclaimed all participation in the wild reveries of the modern revivers of exploded doctrines.

As far as the quarantine laws are concerned, we have always been of opinion that they require revision. The precautions, however, which are taken in other European countries, will not permit us to relax, in

* Med. and Phys. Journal, June 1825, p. 449.

any great degree, without subjecting our commerce to more inconvenience than at present exists. While we profess our firm belief that the plague is contagious, whatever may be its original or annual source, we think the probability of its reaching these shores, or spreading here (should its fomites be imported) is small indeed. At the same time, we quite agree with Mr. Canning that, "if experiments as to non-contagion must be made, he would rather have them made *in corpore vili*, (as for instance on Dr. Maclean himself) than upon the interests and commercial pursuits of a nation."—*Speech in the House of Commons, June 3d*, 1825.

---

3. *Senatus Academicus Edinensis.* This body has altered the statutes respecting the degree of M. D. in the modern Athens. Nor do we think they are altered for the worse. On the contrary, we consider the alteration as a decided improvement. One of the alterations—that of examining the candidate in English, after first testing his classical acquirements by proper classical examination, is what we have preached for many years past—and we maintain that it is the only plan by which a proper estimate can be formed of the said candidate's *classical* acquirements and *medical* knowledge—the true and only objects of the examination. The thesis is still to be *done* in Latin—and that may be all right. The period of study, in some regular university, is extended to four, instead of three years, one of which must be spent, of course, in Edinburgh. Attendance on the following classes is now imperative, viz. anatomy and surgery—chemistry—materia medica and pharmacy—theory of medicine—practice of medicine—midwifery and diseases of women and children—clinical medicine. Two or more of these branches must be attended for six months in each year. Botany three months. The candidate must also attend a course of practical anatomy (dissection)—natural history—medical jurisprudence—clinical surgery—military surgery—and an *additional* hospital attendance of six months in Edinburgh or elsewhere, during two of the four years.

What will the "Society of Physicians," (composed, let it be remembered, of about half a dozen of physicians, who modestly style themselves the representatives of the "peerage of the profession"—risum teneatis?) say to the regulation of *their* alma mater, which now *enjoins* the study of that very branch of the profession, the practice of which excludes them for ever from—the "Peerage!"—Into what a predicament have they thrown themselves by their overweening pride and illiberal prejudice! But why waste words on an association which will never enrol among its members a single physician of respectability, beyond the number composing the original junto.

We cordially agree with the Senatus Academicus in enforcing *the study of all the branches* abovementioned. Surgery and midwifery ought to be studied by the physician, in the same manner and for the same reasons, as physic is studied by the surgeon and the accoucheur. Although, in great cities and large provincial towns, it may be *convenient*

to divide the *practice* of the profession into distinct branches, the *study of the whole* never can be dispensed with, if we wish to see the science advance and impress the public with respect for its professors. It is absurd to think that surgery can be properly practised without a knowledge of the general principles that guide the physician—nor can the physician be competent to treat constitutional diseases without a proper knowledge of the local diseases on which they often depend, or by which they are so generally influenced. Yet, this last class of affections is considered the exclusive property of the surgeon. Again, how can the diseases of parturient women be properly managed by the physicians, without attending to, and carefully studying, that important process of nature with which those diseases are so intimately connected, or on which they so often immediately depend?—The supposition is absurd. The public as well as the profession feel the absurdity of the unnatural disjunction, (as far as study is concerned) and the consequence is very properly a revision and alteration of existing statutes.

P. S. We forgot to mention, that one of the four year's study is allowed to be spent in a university, or "*elsewhere* (for instance London) *provided that the student, in the latter case, attend for six months or more, the practice of some great hospital.*" It is mentioned in the statutes, that the hospital must contain 80 or more beds. A year's grace out of the four is also allowed to medical officers in the army, navy, and East India Company's service.

---

4. *Variolo-Vaccination.** Dr. Ferguson, a young physician of considerable promise, has addressed a letter to Sir Henry Halford on the subject of modified small pox artificially induced by inoculating with both fluids at nearly the same time. The proposition is not entirely novel. Dr. Woodville, in 1799, vaccinated 510 patients *in the small pox hospital*, but, to his astonishment, he found that in the greater number of these, a general eruption of "variolous-like pustules" broke out, instead of the vaccine pustule. Dr. Jenner being acquainted with this event, gave it as his opinion that the vaccine matter must have been *variolated* by the atmosphere of the hospital. In fact, there can be little doubt that this eruptive disease was no other than the modified small pox. Dr. Woodville mixed the variolous and vaccine matter and inserted it into the arms of twenty-eight persons. In one half of these the small pox, and in the other half, the vaccine was produced—"but in none was there many pustules, or much indisposition." It also appears, says Dr. Woodville, "that if the cow-pox matter and the small-pox matter be both inserted in the arm of a patient, even within

* A letter to Sir Henry Halford, Bart. proposing a method of inoculating the small pox, which deprives it of all its danger, but preserves all its power of preventing a second attack. By R. Ferguson, M. D. Member of the Royal College of Physicians of London and Edinburgh. 8vo. stitched pp. 38. London, 8th June, 1825.

an inch of each other, so that at the 9th day the same efflorescence becomes common to both the local affections, nevertheless inoculating from the cow-pox tumour, the genuine vaccine disease will be produced." There are, therefore, four modes of producing the varioloid disease. 1st. By inoculating with both poisons, but in separate places. 2nd. By exposure to a variolous atmosphere and then vaccinating. 3dly. By propagating the varioloid pustule. 4th. By mixing the two poisons together and inserting the compound. The first mode, Dr. Ferguson considers as the best. The second is not to be recommended. The third is dangerous. The fourth is very uncertain. The following are Dr. Willan's conclusions on the combined inoculation of the variolous and vaccine fluids.

"'1. That when a person was inoculated with vaccine and variolous matter, about the same time, both inoculations proved effective; for the vaccine vesicle proceeded to its acmè in its usual number of days, and the maturation of the variolous pustule was attended by a variolous eruption on the skin.

"'2. That these effects took place without much variation, in all cases where the interval between the two inoculations *did not exceed a week*: but,

"'3. That when variolous matter was inserted on the ninth day after the vaccine inoculation, its action seemed to be wholly precluded.'—Willan on Vaccine Inoculation, p. 1." 13.

From Dr. Woodville's experiments, it appears that the modified small pox alluded to, is as perfect a security from future attacks of small pox, as the natural or inoculated small pox itself.—And Dr. Willan concludes "that variolous and vaccine virus inoculated at the same time (not mixed) restrain the operation of each other on the body, and somewhat alter the form of the pustules and vesicles, without effecting any change in the qualities of the fluid they contain." And farther on he observes, that persons affected with eruptions on this plan, "are as safe as the inoculated small pox can render them."

Dr. Ferguson anticipates some objections that may be made to his proposal. "It may be asked," says he, "if small-pox after vaccination be so mild a disease, why not let the patient have the full benefit of vaccination, and give him the chance of escaping the varioloid disease altogether? For if he should take the small-pox, you allow it will be mild.

"In answer to this I say, that though it is generally mild, it may be severe. If not severe enough to kill, yet sufficiently so to disfigure. It may be not at all modified, if the vaccine matter has ceased to influence the constitution: *a case of pure small-pox, therefore, caught naturally!* Now the advantages of inoculating so as to produce this disease will readily appear if it be considered—

"That it is an acknowledged fact, that inoculation renders the small-pox mild. That it is also an acknowledged fact, that the cow-pox renders the small-pox mild.

"If, then, I produce a mild form of small-pox, by inoculation, and mitigate that mild form, by subsequent vaccination, I ensure a disease, which is as secure as the small-pox, and as mild as the chicken-pox."

Another objection is—the forming a source of contagion by this process. Dr. Ferguson does not, we think, appreciate sufficiently the value of this objection. He thinks that, so long as no law exists to enforce vaccination, the carelessness of some and the prejudices of others, will always ensure the existence of small pox. This may be true; but will not the sources be *more numerous* in the one case than in the other? There is one argument, however, in favour of *variolo-vaccination*, which Dr. F. has not adduced. The prejudices against vacination are certainly great among the lower, and the doubts are strong among the higher, orders of society. The most prejudiced and the most sceptical, we think, would not object to the double process, which would appear to them as making security more sure. If this plan is to be acted on, the inoculation of small-pox matter should take place a few days after the vaccination—that is, as soon as it appears that the vaccine matter is taking effect. For if we inserted both fluids at the same time, we might sometimes have small pox without the modifying influence of vaccina.

Dr. Ferguson writes well. He is a gentleman of superior education, whose mind is well stored with the science and literature of his own country, and expanded by travelling abroad.

"Et mores hominum multorum vidit et urbes."

---

5. *Abernethy* versus *The Lancet.* This long-pending and expensive suit has, at length, terminated. The arguments, on both sides, were sustained with all the eloquence, subtlety, and ingenuity, which forensic talent could bring forth. The judgment delivered by the Chancellor on Friday the 17th June, we shall copy from the Courier of that day, and record it without a comment.

"The Lord Chancellor having declared his intention of giving judgment in this important cause, the Court at an early hour was crowded. His Lordship on entering the Court commenced his judgement. This was a motion, he observed, for an injunction to restrain the publishers of the Lancet from communicating to the world the lectures of Mr. Abernethy, and had been argued on three grounds:—A violation of literary property; a breach of trust; and of implied contract. At the conclusion of his former observations, he raised the questions whether, if these publishers were pupils, they would not be guilty of a breach of an implied contract in publishing to the world lectures which it was intended never should be so published; or, if they were not pupils, but obtained their information through some one or other of the pupils, they would not be guilty of such a violation of trust, as would warrant the interference of that Court to the extent now prayed; the importance of the question had led him to advise with the Common Law Judges on the subject; who were unanimously of opinion, that if the publication were by a pupil, an action for damages would lie against him, as the common law would assume an implied contract; or, that if the publication were by a person who received his information through a pupil, and there was no other way in which he could receive it, a similar action would lie against the person thus publishing, for the law would not permit that which could only be obtained by fraud, to be employed by a third party to the injury of the person in whom the property existed. In this view of the law he perfectly concurred; and it was well for the protection of that large and meritorious class of individuals—our public lecturers—that such was the law. On the whole he was of opinion, that it had been made satisfactorily to appear, that this publication was effected by either a breach of implied contract or of trust, and, on either of these grounds Mr. Abernethy was entitled to the injunction prayed.—Motion granted."—*Courier*, 17*th June*, 1825.

